# An Empirical Study on the Effect of Blended Scents in Driving Environments From a Neuro‐Cognitive Perspective

**DOI:** 10.1002/brb3.70082

**Published:** 2024-10-08

**Authors:** Tan Li, Hua Sun, Mianjie Wang, Weihui Dai, Xuesheng Qian

**Affiliations:** ^1^ Shiseido China Innovation Center Shanghai China; ^2^ Shanghai China‐norm Quality Technical Service Co., Ltd Shanghai China; ^3^ Shanghai INEUTECH Technology Co., Ltd Shanghai China; ^4^ School of Management Fudan University Shanghai China; ^5^ Faculty of Innovation Engineering Macau University of Science and Technology Macau China

**Keywords:** aromatherapy, blended scents, cognition, driving environments, EEG

## Abstract

**Background:**

An effective method that is easy to implement and widely applicable for improving driving performance and reducing driving risks remains a challenge. Although fragrances are widely used in daily driving, there is a gap between empirical research on everyday blended fragrances and functional fragrances clinical reports. In this study, a deliberately chosen blend of scent without overtly stimulating or functional proven evidence was tested for its potential to enhance performance in a driving environment.

**Method:**

Thirty qualified young drivers were recruited to participate in the experiment. They were asked to watch a 15‐min first‐person perspective driving video to simulate a driving environment and then complete questionnaires and three sets of behavioral experiments while their brain activity was monitored by EEG.

**Result:**

Participants in the scented environment exhibited statistically significant advantages in two cognitive tasks during behavioral measures. These findings were effectively supported by the EEG data, showing that beta waves exhibited more activity in the occipital and prefrontal cortex, enhanced theta waves were observed in the prefrontal cortex, and the TAB index characterizing driving fatigue was suppressed in the prefrontal cortex.

**Conclusion:**

This empirical evidence highlights the potential of pleasant, natural, and blended scents in enhancing driving performance, suggesting that promoting the aromatherapy while driving as an easily applicable approach in daily life seems justified and expands the application of aromatherapy in daily life.

## Graphical abstract

In the presence of blended pleasant fragrances, participants demonstrated significantly better performance in driving simulation, indicating the potential of aromatherapy to enhance driving performance and reduce driving risks.

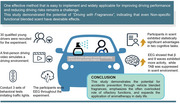

## 1 Introduction

Road traffic accidents have emerged as a significant social problem, posing threats to both lives and property. According to the World Health Organization (WHO), ∼1.3 million people lose their lives each year due to road traffic crashes. Moreover, road traffic injuries are the leading cause of death among children and young adults aged 5–29 years (World Health Organization 2022).

Drivers play a crucial role in road traffic, and their driving performance significantly contributes to traffic accidents. Research has shown that driving while fatigued (Smith 2016), distracted driving (García‐Herrero et al. 2021), and road rage (Sansone, Lam, and Wiederman 2010) are all significant causes of road traffic accidents. Researchers and the industry have been exploring various methods to enhance driving well‐being and performance. These include practices such as listening to music (van der Zwaag et al. 2011), incorporating more rest breaks (Chen and Xie 2014), utilizing in‐vehicle intelligent information technologies (Ward and Hirst 1997), and implementing warning pavement markings (Wood and Donnell 2020). However, these methods have limitations and unexpected negative reports. For example, the complexity and uncertainty of emotional stimulation through music are evident, including the song stimuli varying in valence, to a lesser extent in energy levels, as well as significant individual differences (van der Zwaag et al. 2011; Sloboda and Juslin, 2010). Additionally, increasing rest time is not always cost‐effective for safe driving, as it involves multiple factors. A truck driver who takes three rest breaks may end up more fatigued than one who takes once or twice (Chen and Xie 2014). Furthermore, there is fatigue unrelated to sleep (Saxby et al. 2013). Therefore, determining the most effective duration and timing of rest breaks remains a challenge that needs further investigation. The adaptive capabilities of intelligent information systems in vehicles need improvement to avoid being perceived as redundant additions to the transportation system. The establishment and maintenance of traffic warning signs are not uniformly consistent worldwide. In summary, finding an ideal method that is easy to implement and widely applicable to maintain drivers’ well‐being while driving remains a challenge. Further research and development are needed to identify effective strategies that can be easily adopted and promote the performance and well‐being of drivers on the road.

Hence, the prevalent notion that driving safety is solely seen as a singular phenomenology matter is inadequate. This implies that the measures taken to only address the phenomenon are not sufficient. Instead, in fact, driving is a cognitive activity with a higher load, requiring full‐body collaboration and human–computer coupling (Palmiero, Piccardi, and Boccia 2019; Ware et al. 2020). It includes forming awareness, judging decisions, regulating attention, and so forth. Therefore, it may be more convincing to explain driving safety from a neuro‐cognitive perspective (Haghani et al. 2021).

There is a close relationship between cognitive and olfaction (Andrew 2011). Through the sense of smell, fragrances are widely recognized to have an influence on human psychophysiology. These have contributed to the popularity of fragrances and aromatherapy, where natural fragrances and aromatic compounds are consciously or unconsciously integrated into folk medicine and daily life (Sowndhararajan and Kim 2016), ubiquitous in today's world. In the field of transportation, fragrances are often spontaneously applied to driving space. Although most car scents are not set for specific purposes, in purposeful studies, the benefits of different scents for driving are being continually discovered. For example, lemon fragrance has been shown to improve certain aspects of driving performance (Baron and Kalsher 1998) and promote better braking (Dmitrenko, Maggioni, and Obrist 2018). Lavender fragrance has been found to improve the driver`s feelings toward calm, comfort, relaxation, and freshness (Mustafa et al. 2016). Peppermint fragrance shows promise as an in‐vehicle scent to sustain drivers’ alertness (Mahachandra, Yassierli, and Garnaby 2015). These findings highlight the potential of fragrance to enhance driving performance and well‐being. Therefore, compared to other types of reminders, scent reminders are considered more comfortable and convenient.

However, the evidence presented above is still insufficient. While extensive research has been conducted on the specific effects of fragrances on human physiological and psychological aspects, driving, as previously mentioned, is a complex cognitive behavior involving a comprehensive human–machine interaction. Instead of preparing a variety of function‐specific scents, akin to medications, in the vehicle to address cognitive or emotional risks during driving, most drivers simply choose scents they enjoy, selecting for personal use rather than specific functional purposes. Hence, it is worth exploring whether fragrances do have a positive cognitive effect during driving and whether the functional evidence of a single fragrance can be generalized.

Drivers' behavioral inhibition and driving self‐regulation mediated the effect of attention impulsivity on driving errors. Executive functions, reflective functioning, and driving self‐regulation mediated the relationship between motor impulsivity and driving errors (Memarian et al. 2023). Therefore, this study devised questionnaires and three sets of behavioral experiments with different cognitive emphases to test the hypothesis that comprehensive aroma is beneficial to driving performance, while continuously monitoring brain activity using electroencephalogram (EEG) for the exploration of neurophysiological mechanisms.

## 2 Materials and Methods

### 2.1 Participants

A total of 32 participants, each possessing a driver license and over 2 years of driving experience, and commuting by car almost daily in Shanghai, voluntarily engaged in the experiment. These participants met specific inclusion criteria, including normal or corrected‐to‐normal vision, absence of color blindness, right‐handedness without acquired motor impairments, intact olfactory function, and the absence of olfactory impairments caused by conditions such as nasal congestion (e.g., colds) or underlying neurological disorders. During the experiment, participants refrained from perfume or other scented cosmetics. Additionally, the participants did not include professional drivers.

Two participants were excluded from the study due to abnormal EEG data caused by unexpected activities, resulting in a final cohort of 30 participants (18 males and 12 females) with an average age of 24.2 years (*SD* = 2.37) and an average driving experience of 37.26 months (SD = 7.90).

All participants gave their informed consent in writing and were financially compensated for their participation. Ethical approval for the study was obtained from the University Ethics Committee (HR 223–2020).

### 2.2 Scent Stimuli

The fragrance material utilized in this experiment was a blend of scents developed by Shiseido. The formulation comprised a mixture of fragrances, with the primary constituents present in concentrations exceeding 3% as follows: jasmine at 26%, musk 13%, muguet at 12%, rose at 12%, ambery at 10%, bergamot at 7%, osmanthus at 4%, and freesia 4%. Notably, the chosen fragrance did not incorporate scents with overtly stimulating properties (such as peppermint or rosemary) or scents that have been definitively validated to possess positive value in driving (such as Lavender or Lemon). This deliberate selection aimed to assess the cognitive significance of daily fragrances rather than singular functional scents due to their relevance to cognition associated with driving inherently.

To simulate the in‐car fragrance setting, the distance between the fragrance release device and the participants’ faces was standardized at 70 cm.

### 2.3 Questionnaires

The questionnaire consisted of three parts: (1) demographic characteristics; (2) odor evaluation: Question 1 validated the perception of the scent, using a seven‐point Likert scale, to distinguish the effectiveness of fragrance stimulation delivery, while Questions 2–4 assessed the polarity of attitudes toward the scent stimuli also using a seven‐point Likert scale; (3) task evaluation: Questions 5–6 evaluated the difficulty of behavioral tasks using a percentage‐based scale, and Questions 7–8 collected self‐subjective task completion satisfaction and fatigue levels, as shown in Table 1.

**TABLE 1:** Questionnaires.

Dimension	Question	Method
Odor evaluation	(1) I can feel this smell	Seven‐point scale
	(2) The smell makes me happy	Seven‐point scale
	(3) The smell makes me relax	Seven‐point scale
	(4) Overall, I like the smell	Seven‐point scale
Task evaluation	(5) The mental pressure of tasks (0–100)	Percentage‐based scale
	(6) The pressure of tasks in terms of deadlines (0–100)	Percentage‐based scale
	(7) Satisfaction level of task self‐expression (0–100)	Percentage‐based scale
	(8) I feel the level of fatigue now (five‐point scale)	Five‐point scale

### 2.4 Behavioral Tasks

The study featured three distinct task types: oddball (Delgado et al. 2020), go/nogo (Criaud and Boulinguez 2013), and Stroop (Yuan et al. 2020) tests. Among these, considering measures of vigilant attention and fatigue, the go/nogo task incorporates elements of psychomotor vigilance test (PVT) that is the simple reaction time (RT) to stimuli that occur at random intervals (Basner, Mollicone, and Dinges 2011). Traffic signal elements were incorporated in the tasks to align with the driving environment. The above three tests all have minimal learning effects, minimizing the variability due to participants’ different abilities and experiences. The rationale behind utilizing these three distinct behavioral tasks was to comprehensively investigate the impact of different odor environments on drivers' inhibitory abilities, sustained attention performance, and cognitive conflict from multiple perspectives. Task programming was executed using E‐prime (Version 3.0), and task durations varied due to differences in participants' response times, averaging ∼5 min per task.

**FIGURE 1:**
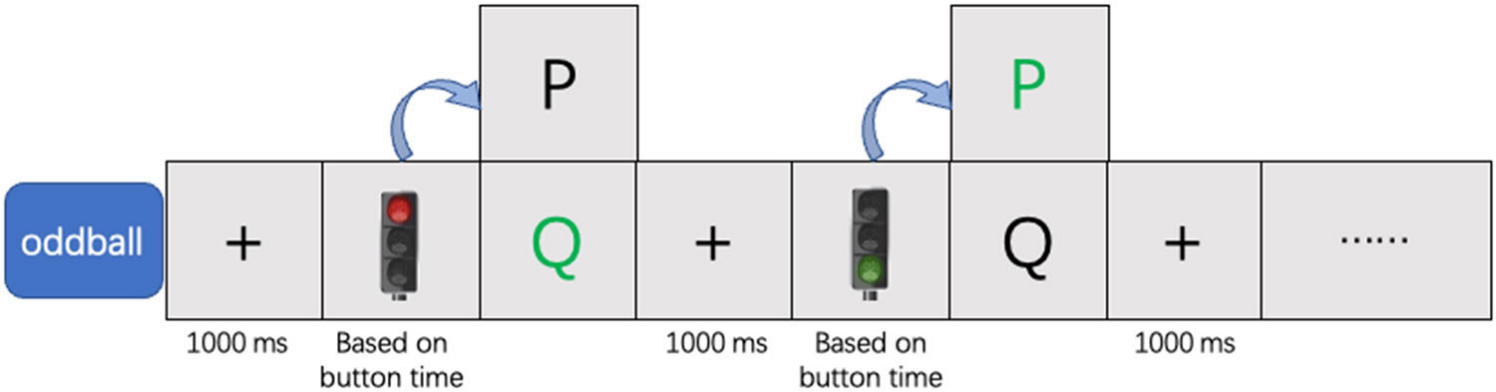
Experimental design of Task 1.

Task 1: The oddball task encompassed two categories of stimuli: infrequent deviant stimuli (green lights with a 20% occurrence rate) and frequent standard stimuli (red lights with an 80% occurrence rate). Participants were required to produce two distinct responses to these stimuli: one for standard stimuli (keypress “Q”) and another for deviant stimuli (keypress “P”) (Figure 1). Consequently, responses to the more frequently occurring standard stimuli were classified as dominant responses, while responses to the less frequent novel stimuli were categorized as non‐dominant responses. To ensure accurate responses to novel stimuli, participants had to inhibit their dominant responses to standard stimuli upon detecting the presence of novel stimuli. In doing so, we were able to measure individuals' behavioral inhibitory capabilities.

**FIGURE 2:**
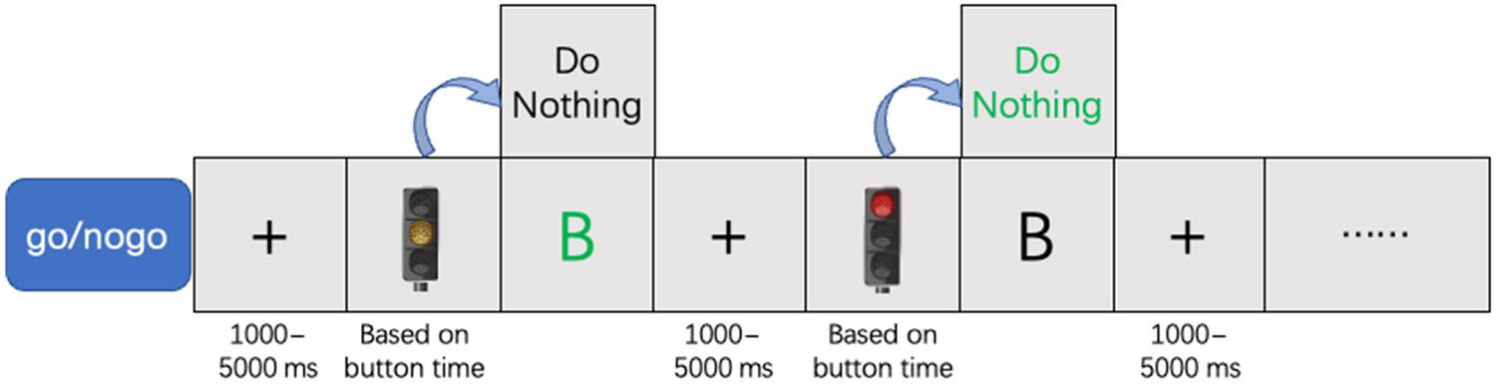
Experimental design of Task 2.

Task 2: In the “go/nogo” task, the screen presented two types of traffic experimental stimuli: yellow lights (occurring 18 times) and red lights (occurring two times). Meanwhile, the unpredictability of the stimulus intervals in the PVT paradigm, which has been used in fatigue driving (Gibbings et al. 2022), is incorporated into the task. Participants were instructed to respond rapidly and accurately (by pressing the “B” key) to the yellow light (GO) stimuli while refraining from making any response to the red light (NOGO) stimuli presented during the course of the experiment, with randomized time intervals ranging from 1 to 5 s (Figure 2). This task was employed to evaluate participants' executive function and sustained attention capabilities.

**FIGURE 3:**
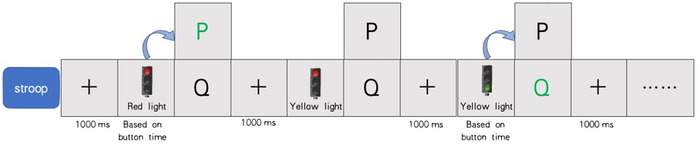
Experimental design of Task 3.

Task 3: In the Stroop task, participants were presented with a sequence of traffic signals accompanied by descriptive words. These words could either be congruent with the meaning of the signal (e.g., a red signal paired with the word “red”) or incongruent (e.g., a green signal paired with the word “yellow”). Participants were tasked with evaluating the congruence between the descriptive word and the signal and responding accordingly. If they perceived the meaning of the red signal to be congruent, they were instructed to press the “P” key; conversely, if it was incongruent, they were instructed not to press the key; if a different signal appeared, they were to press the “Q” key (Figure 3). This task established conditions of cognitive congruence and cognitive conflict based on the alignment between the descriptive word and the signal, allowing us to assess individuals' capacity to manage cognitive conflicts.

### 2.5 Experimental Procedure

**FIGURE 4:**
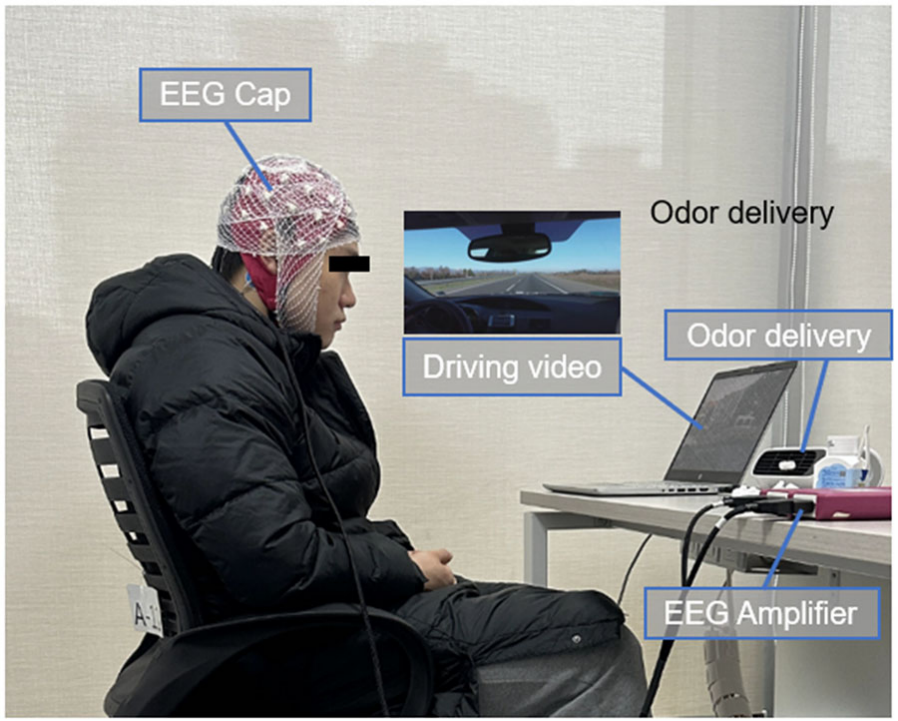
Experiment environment.

1. Participants guidelines: Before participating in the tests, participants were instructed by the staff not to consume coffee, alcohol, nicotine, or any other psychoactive substances on the day prior to and the day of the tests. Additionally, they were advised not to apply perfumes or use scented cosmetics, shampoos, and so forth. The experiments were conducted between 10 a.m. and 6 p.m. as this time frame is considered to have stable blink rates and less susceptibility to diurnal variations in the peak of sleepiness.
2. Experiment preparation: Prior to the formal commencement of the tests, preparations were made for the olfactory stimuli, including scent diffusers, scent sticks (designated as scent stick 1—odorless, scent stick 2—odor), and other necessary equipment. Scent sticks were prepared by the staff members following standard procedures and placed it on an odor delivery that simulates a car vent.
3. Participants preparation: Participants began by signing informed consent forms. Following that, they completed self‐assessments to evaluate their levels of fatigue and subjective odor preferences. Subsequently, they proceeded with the collection of resting‐state EEG data as baseline. Afterward, they spent ∼10 min in a waiting room, engaging in practice sessions to familiarize themselves with the procedures of the behavioral experiment. This ensured that participants were proficient in the behavioral experiment operations and minimized the potential interference of learner effects on the results as much as possible. Considering the possible order effects, participants were randomly divided into two groups on average: odor to odorless and odorless to odor.
4. Behavioral pre‐task: Grouped participants needed to complete three sets of behavioral tasks. During the tasks, software recorded participants' judgment operations and RTs as baselines of behavioral tasks.
5. Driving simulation: During this phase, participants were exposed to a driving scene simulation while wearing EEG equipment. The simulation was watching a 15‐min driving video from a first‐person perspective, aiming to replicate the cognitive state experienced during actual driving. Prior to starting the video, participants were instructed to minimize head and body movements, avoid prolonged eye closure, and maintain silence (Figure 4). To ensure full cognitive engagement, participants were encouraged to pay close attention to the road conditions portrayed in the video, as they would be required to answer relevant questions after the experiment.
6. Behavioral post‐task: After the simulation, participants once again completed the self‐assessment of fatigue levels. Subsequently, participants needed to complete three sets of behavioral tasks once again. Note that the scent still existed in the odor round.
7. Second round: After completing the previous process, participants were provided with a 30‐min rest period. During this time, the laboratory was adequately ventilated to ensure the absence of any lingering odors. Subsequently, the researchers replaced the scent stick and provided guidance to the experimenters for conducting a second round of experiments.

**FIGURE 5:**
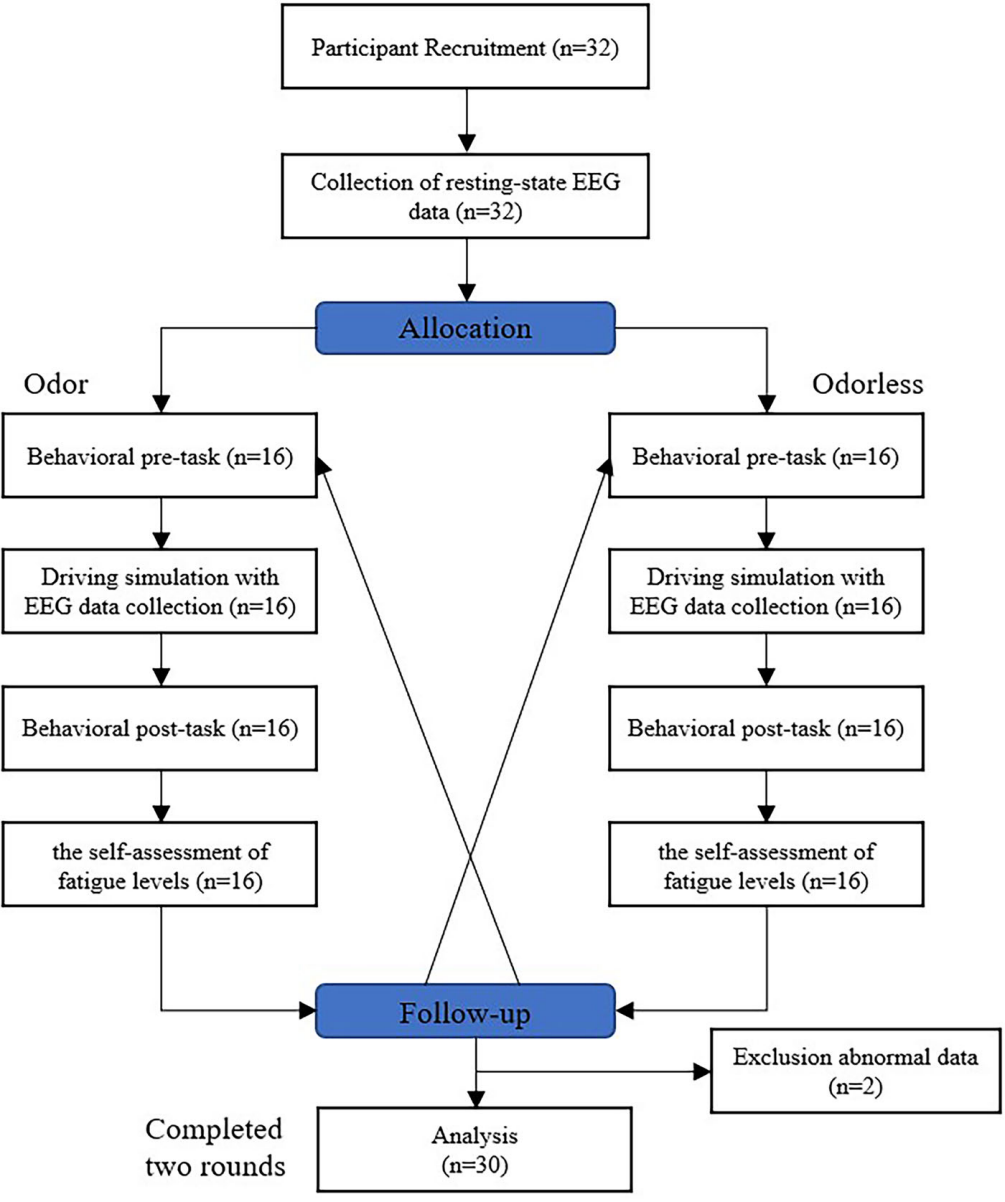
Experimental procedure.

The experimental procedure described above is depicted in Figure 5.

### 2.6 EEG Data Acquisition

**FIGURE 6:**
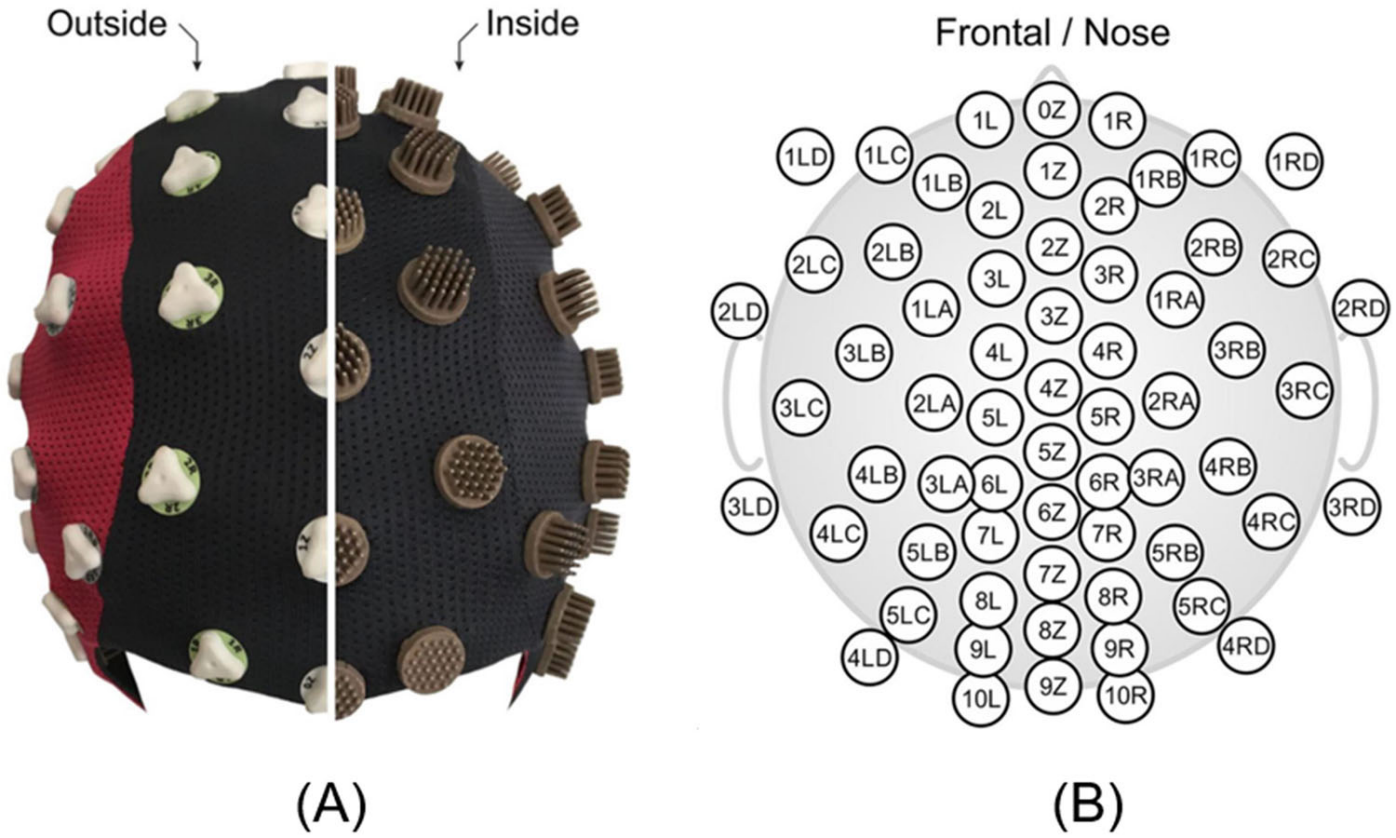
EEG cap and placed electrode distribution.

The EEG system used eego mylab (ANT Neuro, Netherlands) by 64 channels with 1000 Hz sampling frequency, and a multi‐channel dry electrode cap (Waveguard touch CY‐261; ANT Neuro) was utilized. As illustrated in Figure 6, all electrodes were integrated into a flexible fabric cap and arranged in an equidistant layout. The EEG metrics were sampled at 1000 Hz.

The EEG cap depicted in Figure 6: Figure 6A shows a 64‐channel dry PU‐AgCl multi‐pin electrode cap with an equidistant layout, with the left side showing an outside view and the right side showing an inside view. The corresponding detailed 2D electrode topological layout is shown in Figure 6B for the dry cap.

### 2.7 EEG Data Preprocessing

MATLAB R2019b with the eeglab 2019_0 plugin was employed for EEG data preprocessing. The processing of the EEG signal involves applying a high‐pass filter to eliminate frequencies below 0.5 Hz and a low‐pass filter to remove frequencies above 30 Hz. Subsequently, four fundamental EEG frequency patterns are extracted: delta (0.5–4 Hz), theta (4–8 Hz), alpha (8–13 Hz), and beta (13–30 Hz).

For handling blink artifacts, the study used the eeglab plugin's run independent component analysis (ICA) to process the filtered data segments, resulting in improved EEG signal quality.

*z*‐Score calculations were then applied to the EEG data in different frequency bands for subsequent analysis.

### 2.8 Data Statistics

#### 2.8.1 Questionnaire Analysis

The questionnaire data were analyzed using IBM SPSS v.26 (IBM Corp., USA). For Questions 2–4, which have directional consistency, a test of reliability and validity was conducted. Specifically, the Cronbach's *α* for that was .973, with a KMO value of 0.786, and the Bartlett sphericity test reached significance (*p* < .001). A series of *t*‐tests were then conducted on the questionnaire results to analyze the differences between pre‐ and post‐tests as well as different rounds, and Bonferroni correction was used for significant results.

#### 2.8.2 Behavioral Data Analysis

In the behavioral experiment analysis, IBM SPSS v.26 (IBM Corp.) was used for data preprocessing, analysis of variance (ANOVA), and for conducting Bonferroni correction on the statistical results.

For each participant, to ensure effective data analysis, data points for RTs in three types of tasks (oddball task, go/nogo task, and Stroop task) that exceeded the mean ± 3 standard deviations (SD) were excluded. This point primarily focused on participants' RTs and accuracy in behavioral analysis, simultaneously examining the effects of gender and order on the results.

#### 2.8.3 EEG Analysis

To investigate the odor or odorless environments affecting on participants, univariate ANOVA was conducted on the *z*‐score values of power spectral density in different EEG signal frequency bands. Categories (resting state, odorless, and odor), frequency bands (alpha, beta, theta, delta, and TAB—(alpha + theta)/beta), and brain regions (central area, frontal lobe, occipital lobe, parietal lobe, prefrontal cortex, and temporal lobe) were used as within‐subject factors to examine the differences in EEG wave feedback under different conditions. Bonferroni corrections were conducted on the significance test. All the analyses mentioned above were carried out using IBM SPSS v.26 (IBM Corp.), and *p*‐values less than .05 were considered statistically significant.

Lastly, to visualize the EEG wave feedback in different brain regions of participants under different odor environments more clearly, this study employed the Python mne library to create topographical brain maps of power spectral density *z*‐score values, presenting the results graphically.

## 3 Result

### 3.1 Questionnaires

The questionnaire results are shown in Table 2 and the statistical test results in Table 3.

**TABLE 2:** Questionnaire results.

		Odorless (MN ± SD)		Odor (MN ± SD)	
*N* = 30		Pre‐test	Post‐test	Pre‐test	Post‐test
Odor evaluation	(1) I can feel this smell	4.60 ± 2.67	4.27 ± 2.50	6.37 ± 1.47	6.37 ± 1.27
	(2) The smell makes me happy	4.80 ± 2.71	4.83 ± 2.53	6.63 ± 1.33	6.27 ± 1.26
	(3) The smell makes me relax	5.10 ± 2.90	5.17 ± 2.89	6.37 ± 1.47	6.27 ± 1.57
	(4) Overall, I like the smell	4.87 ± 2.79	4.67 ± 2.58	6.63 ± 1.56	6.50 ± 1.36
Task evaluation	(5) The mental pressure of tasks	41.43 ± 27.88	41.73 ± 28.75	37.00 ± 27.92	39.40 ± 27.46
	(6) The pressure of tasks in terms of deadlines	37.43 ± 25.16	35.23 ± 23.68	31.87 ± 26.35	36.43 ± 25.59
	(7) Satisfaction level of task self‐expression	36.77 ± 39.08	36.70 ± 36.27	35.07 ± 36.55	37.43 ± 35.60
	(8) I feel the level of fatigue now	1.73 ± 0.78	2.07 ± 0.91	2.03 ± 0.96	2.40 ± 0.97

**TABLE 3:** Questionnaire significance statistics.

		Odorless pre‐test vs. odorless post‐test		Odorless pre‐test vs. odor pre‐test		Odor pre‐test vs. odor post‐test		Odorless post‐test vs. odor post‐test	
*N* = 30		*F*	*p*	*F*	*p*	*F*	*p*	*F*	*p*
Odor evaluation	(1) I can feel this smell	0.248	.62	0.248	.62	0.080	.78	16.766	.00**
	(2) The smell makes me happy	0.002	.96	11.086	.00**	1.208	.28	7.703	.01**
	(3) The smell makes me relax	0.008	.93	0.008	.93	0.065	.80	3.352	.07
	(4) Overall, I like the smell	0.083	.77	0.083	.77	0.124	.73	11.879	.00**
Task evaluation	(5) The mental pressure of tasks	0.002	.97	0.002	.97	0.113	.74	0.103	.75
	(6) The pressure of tasks in terms of deadlines	0.122	.73	0.000	.99	0.464	.50	0.036	.85
	(7) Satisfaction level of task self‐expression	0.000	.99	0.030	.86	0.065	.80	0.006	.94
	(8) I feel the level of fatigue now	2.316	.13	1.746	.19	2.159	.15	1.893	.17

*p < .05; **p < .01.

In odor evaluation, there was a statistically significant difference between odorless and odor in the post‐test about question (1)—“I can feel this smell” (*F*(1,58) = 16.766, *p* < .001) that indicated the odor stimulation can be perceived. Meanwhile, the odor reflected a positive subjective response which showed a significant statistical difference in the post‐test about questions (2) and (4).

In task evaluation, there was no statistically significant difference in task evaluations among participants between odor and odorless. In addition, there was also no statistical significance in gender and order effect.

### 3.2 Behavioral Tests

**FIGURE 7:**
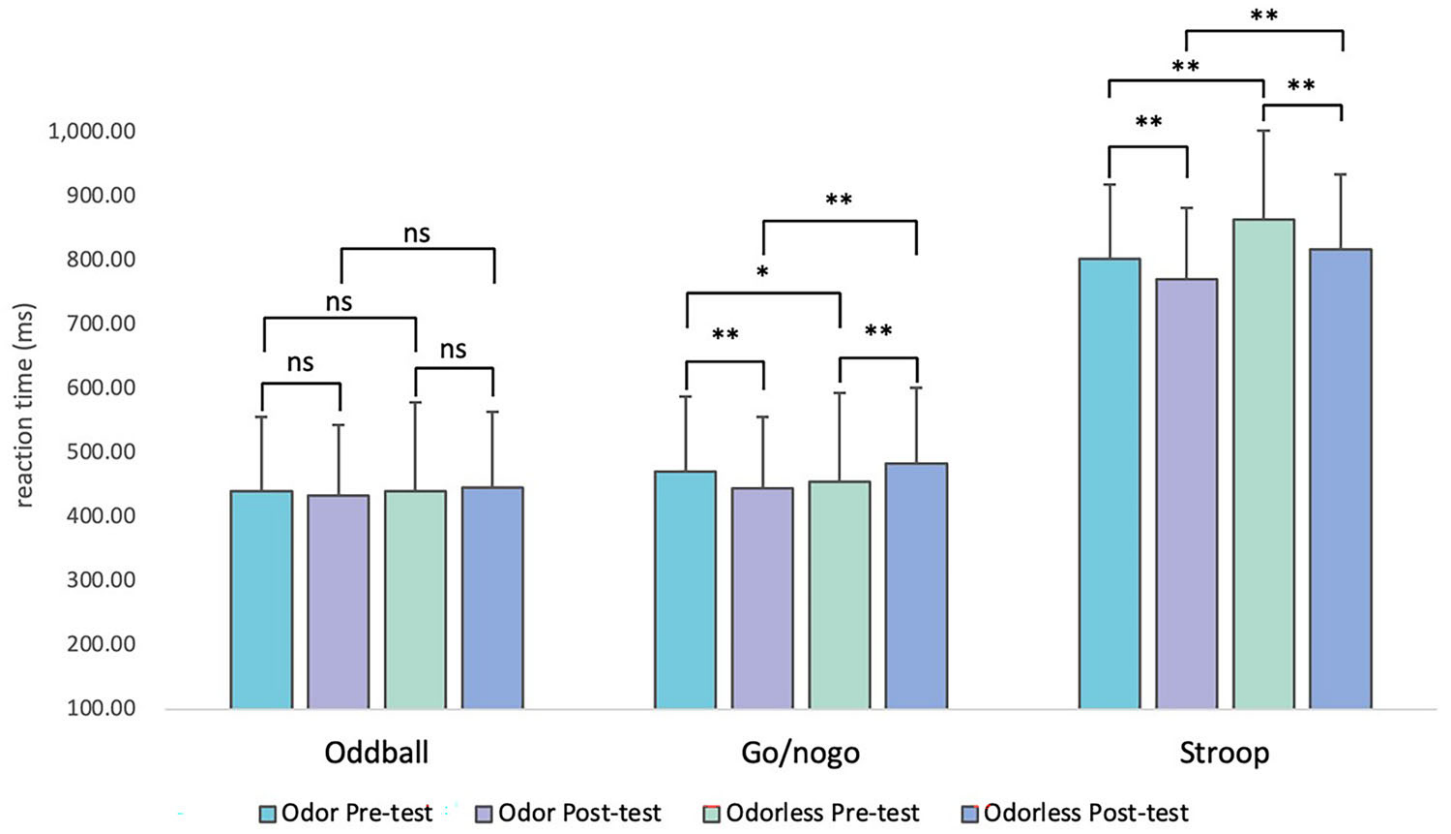
Behavioral mean RT and statistical significance analysis. ns, no significance. *p < .05; **p < .01.

Due to the lack of statistically significant differences in accuracy among participants in different scent environments, the analysis focused primarily on the participants’ RT, as shown in Tables 4 and 5 and Figure 7. In the odorless environment, the post‐test RT for the oddball and go/nogo tasks was higher than the pre‐test. Specifically, the go/nogo task showed statistical significance (*F*(1,58) = 12.367, *p* < .001), indicating the impact of simulated driving environment on participants’ attention and executive function.

**TABLE 4:** Results of behavioral RT.

	Odor (MN ± SD)		Odorless (MN ± SD)	
*N* = 30	Pre‐test	Post‐test	Pre‐test	Post‐test
Oddball	440.90 ± 98.46	433.34 ± 67.51	440.50 ± 80.14	446.50 ± 78.63
Go/nogo	471.76 ± 124.17	445.49 ± 114.06	455.65 ± 113.48	483.85 ± 136.56
Stroop	803.28 ± 169.60	771.57 ± 165.83	863.92 ± 193.97	817.37 ± 174.71

*p < .05; **p < .01.

**TABLE 5:** Statistics of odorless versus odor behavioral RT.

	Odorless pre‐test vs. odorless post‐test		Odorless pre‐test vs. odor pre‐test		Odor pre‐test vs. odor post‐test		Odorless post‐test vs. odor post‐test	
*N* = 30	*F*	*p*	*F*	*p*	*F*	*p*	*F*	*p*
Oddball	0.247	.62	0.004	.95	0.478	.49	2.027	.16
Go/nogo	12.367	.00**	4.353	.04*	11.833	.00**	22.990	.00**
Stroop	20.996	.00**	36.294	.00**	12.255	.00**	24.289	.00**

*p < 0.05; **p < 0.01.

In contrast, in the odor environment, the post‐test RT for all three tests was lower than the pre‐test, with the go/nogo task (*F*(1,58) = 11.833, *p* < .001) and Stroop task (*F*(1,58) = 12.255, *p* < .001) showing statistical significance.

In addition, in all three sets of tasks, the post‐test RT with odor rounds was also lower than that odorless rounds. Tasks go/nogo (*F*(1,58) = 22.990, *p* < .001) and Stroop (*F*(1,58) = 24.289, *p* < .001) demonstrated statistical significance, indicating the enhancing effect of olfactory environment on cognitive function.

Unexpected, the post‐test RT for the Stroop task in odor round was statistically significantly lower than the pre‐test, indicating that this test may have a certain degree of learning effect though pre‐practice had been conducted.

### 3.3 Electroencephalogram

**FIGURE 8:**
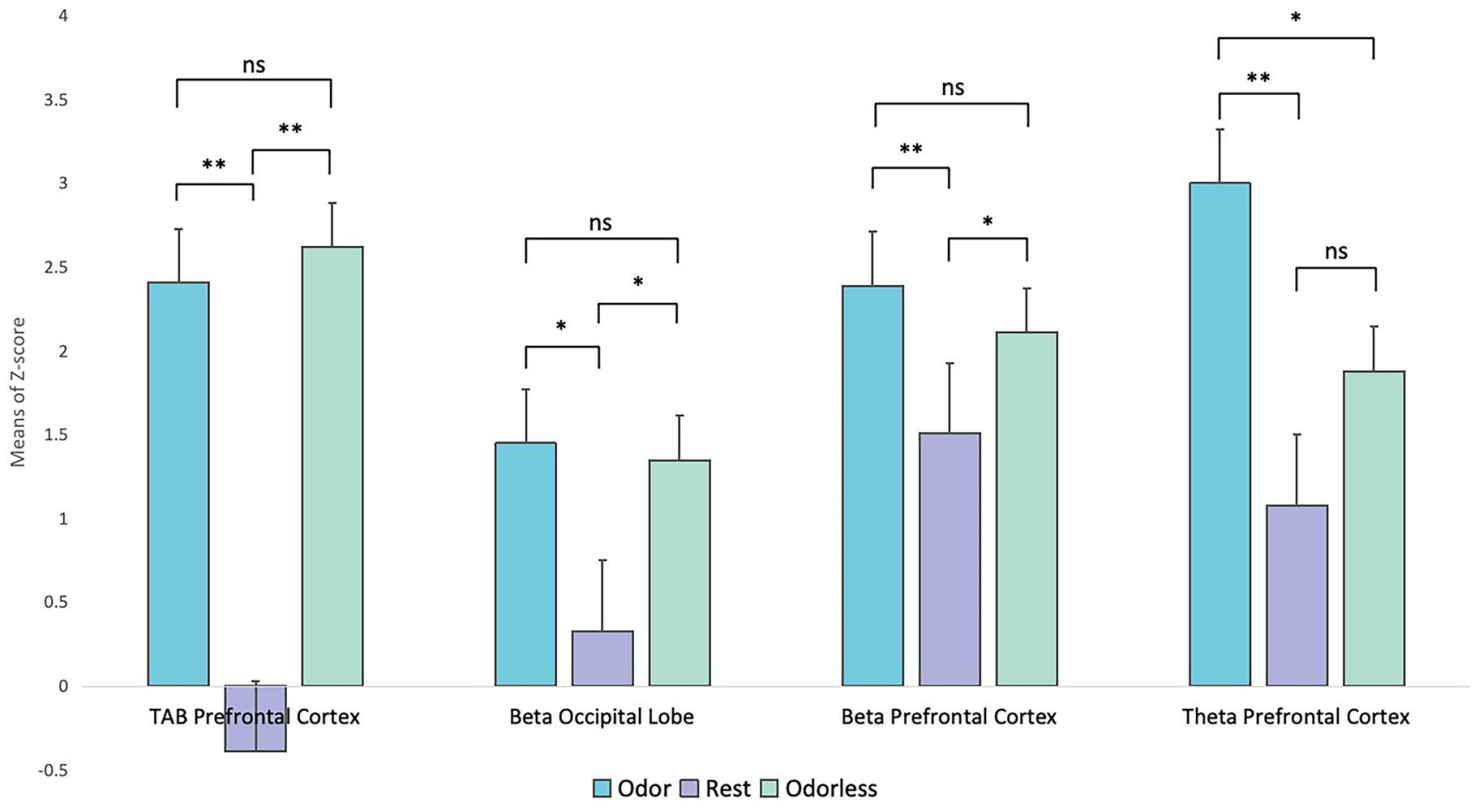
Result of EEG recording and statistical significance analysis. ns, no significance; *p < .05; **p < .01.

The EEG recording, as presented in Table 6 and Figure 8, revealed statistically significant differences in the *z*‐scores of the TAB index within the prefrontal region between the resting state and driving simulation execution, both in the presence of odor (*F*(1,4) = 104.777, *p* < .001) and in an odorless environment (*F*(1,4) = 137.987, *p* < .001). Moreover, the *z*‐score under odor in the prefrontal region was lower than that under odorless conditions.

**TABLE 6:** Result of EEG recording.

				State			
				Odorless		Rest	
Index	Location	State	MN ± SD	*F*	*p*	*F*	*p*
TAB	Prefrontal Cortex	Odor	2.41 ± 0.41	0.449	.641	104.777	.000**
		Rest	−0.39 ± 0.25	137.987	.000**	—	—
		Odorless	2.62 ± 0.37	—	—	—	—
Beta	Occipital lobe	Odor	1.45 ± 0.58	0.027	.826	8.092	.014*
		Rest	0.33 ± 0.36	3.501	.026*	—	—
		Odorless	1.35 ± 0.88	—	—	—	—
	Prefrontal Cortex	Odor	2.39 ± 0.92	0.172	.549	4.738	.007**
		Rest	1.51 ± 0.34	4.924	.036*	—	—
		Odorless	2.11 ± 0.67	—	—	—	—
Theta	Prefrontal Cortex	Odor	3.00 ± 1.07	2.601	.015*	7.593	.000**
		Rest	1.08 ± 0.56	3.136	.080	—	—
		Odorless	1.88 ± 0.55	—	—	—	—

*p < .05; **p < .01.

Similar results were observed in the *z*‐scores of the beta waves in the prefrontal region, showing significant differences between the resting state and simulation execution in both the odor (*F*(1,4) = 4.738, *p* = .007) and odorless (*F*(1,4) = 4.924, *p* = .036) conditions. In beta *z*‐scores within the occipital region, significant differences were observed during simulation execution, manifested as odor (F(1,4) = 8.092, *p* = 0.014) and odorless (F(1,4) = 3.501, *p* = 0.026). Whether in the occipital lobe or the prefrontal region, the z‐score of beta waves in an odorous environment was higher than in an odorless environment.

In theta *z*‐scores within the prefrontal region, these differences were evident when comparing odor to the resting state (*F*(1,4) = 7.593, *p* < .001) during simulation execution and resting. In contrast, the difference between odorless and the resting state was not significant (*F*(1,4) = 3.136, *p* = .080). Furthermore, significant differences were observed between the odor and odorless conditions (*F*(1,4) = 2.601, *p* = .015).

## 4 Discussion

### 4.1 The Effect of Fragrance on Cognition

Driving is a complex cognitive task that requires sustained and appropriate attention engagement (Kerruish et al. 2022). This study substantiates that driving fatigue can be easily triggered, especially in the context of prolonged monotonous activities that a 15‐min first‐person monotony driving video is sufficient to elicit significant neural signals of fatigue, as indicated by the TAB index, commonly used to reflect driving fatigue (Jap et al. 2009; Xavier, Su Ting, and Fauzan 2020; Puspasari et al. 2023), significantly observed in the prefrontal cortex.

While previous research has not explicitly emphasized the difference between fatigue and boredom (Lal and Craig 2001), what can be confirmed is that repetitive operations, such as repetitive visualization, can be significant contributors to driving fatigue. Previous research has demonstrated that in laboratory studies involving monotonous and boring tasks, declines in cognitive performance can occur in as little as 10 min, and these decrements further increase over time (Caldwell et al. 2019). The fatigue‐inducing effects of repetitive operations, including repetitive visual stimuli and mechanical movements, have been the focus of numerous studies (Borghini et al. 2013; Di Stasi et al. 2013; Jiang et al. 2024). These scholarly perspectives should also be supported by a wealth of everyday driving experience.

In line with previous evidence of fragrance on cognition, the experiment scent exhibited a positive effect. While participants did not report significant differences in task difficulty in the subjective questionnaires, their performance in the behavioral experiments showed remarkable improvements for participants in the scented environment between pre‐ and post‐tests, in spite of not every item being statistically significant. The results of the behavioral experiments indicated that participants in the scented environment demonstrated better cognitive performance, including higher cognitive conflict processing abilities and better inhibition of negative cognitive effects under fatigue.

Based on EEG observations, we are able to reinforce the above findings that beta waves, indicative of focus and heightened state of awareness, exhibit more activity in the occipital and prefrontal cortex in odor round. Concurrently, the TAB index associated with fatigue and drowsiness is suppressed. These neurological findings provide an explanation for the aforementioned behavioral experimental results.

Theta waves have often been associated with mental fatigue in previous research (Wascher et al. 2014), a state that typically accompanies a decrease in alpha and beta waves (Jap et al. 2009). In contrast, the fragrance itself can evoke theta waves (Sowndhararajan and Kim 2016), representing a state of relaxation, akin to a combined form of Tai‐chi/yoga (Field, Diego, and Hernandez‐Reif 2010), significantly enhancing a relaxed state, which is akin to a deeply relaxed and inward focused state (Abhang, Gawali, and Mehrotra 2016).

### 4.2 The Mechanism of Fragrance on Physiological and Psychological Aspects

The reason for the aforementioned cognitive enhancement comes from the sense of smell and the olfactory system. The olfactory system serves as an ideal model for unraveling the neural mechanisms underlying consciousness (Morsella, Krieger, and Bargh 2010). Compared to the visual and auditory systems, the olfactory system provides a simpler and more practical framework for studying the intricate connection between consciousness and attention (Keller 2011). This allows odorants to influence the sympathetic and parasympathetic nervous systems (Angelucci et al. 2014), as well as neurophysiological brain activity (Keller 2011), potentially affecting not only emotions but also overall bodily function (Denda et al. 2000). Studies have indicated that the olfactory stimulation can lead to immediate alterations in physiological indicators, including blood pressure, muscle tension, pupil dilation, skin temperature, pulse rate, and brain activity (Angelucci et al. 2014; Diego et al. 1998).

More recent studies suggest that the neurocircuit involved in olfactory odor pleasantness processing are overlapped with the attention network in some extent (Ruser et al. 2021). These physiological mechanisms lead to the important role of pleasant odors in human cognition, behavior, and emotion (Holland, Hendriks, and Aarts 2005). Studies also have shown that these scents can positively impact cognition by altering the focus of visual‐spatial attention (Rinaldi et al. 2018), heightening awareness (Shimizu, Gyokusen, and Kitamura 2008), and enhancing the precision of attention or the efficiency of task performance (J. Liu et al. 2019). Contrastingly, while unpleasant odors are rooted in evolutionary necessity and exhibit heightened processing capabilities (Boesveldt et al. 2010), they may exert detrimental influences on cognitive functions. Specifically, they can impair the state of attentiveness and alertness that is crucial for maintaining vigilance (Ruser et al. 2021).

On the other hand, the ancillary effects of mood enhancement due to pleasant scent should also not be overlooked (Grabenhorst, Rolls, and Margot 2011). Elevation in positive mood is linked with improvements in cognition (Isen 2000; Andrew 2011). Considering the positive feedback regarding the scent in the questionnaires, it is possible that the enhancement in cognitive performance can be attributed to the positive emotional response elicited by the odor, leading to a combined effect of improved mood and cognition, including increased tolerance and endurance for tedious and fatigue‐inducing tasks, such as driving.

### 4.3 The Efficacy of Blended Fragrances Versus Specific Functional Scents

Furthermore, attention needed to be given to the blended scents. From the literature of previous studies, research on the psychological and physiological effects of fragrances has focused on single scents (Sowndhararajan and Kim 2016), with lavender, peppermint, and rosemary being the most studied aromatic plants (Ali et al. 2015). However, to the scent stimuli used in this study, the scents mentioned above were excluded intentionally in order to eliminate the influence of stimulating properties or specific functionalities on the results.

Regarding the scent of the primary components involved in the experiment, while some fragrances still have been studied in behavioral or neuroscientific research, such as the natural fragrance of jasmine is shown to increase the beta wave activity (Sayowan et al. 2013) and promote positive emotions and relaxation (Xiong et al. 2023); the fragrances of jasmine and rose each have an awakening effect (Klemm et al. 1992); inhaling musk had the effect of relieving stress (Fukui et al. 2007), improving working memory (Hasheminia and Sho'ouri 2023), providing mild sedation and reducing pain (Nascimento et al. 2022); inhaling bergamot has been found to relieve work‐related stress with various workloads (S.‐H. Liu, Lin, and Chang 2013) and induce a relaxation state of brain similar to listening to soft music (Peng, Koo, and Yu 2009).

Apart from these scattered individual pieces of evidence, considering the significant role of fragrance concentration on EEG activity (Sowndhararajan and Kim 2016), and the low concentration of each specific fragrance in this study, we believe that the experimental results are primarily attributed to the overall benefits of blended fragrances, which bring an enhancement in cognitive ability and an implicit sense of mental pleasure.

### 4.4 The Potential Efficacy of Fragrances in Daily Driving

Aromas affect human beings in terms of both physiological and psychological aspects. In traditional medicine as well as in aromatherapy and herbal medicine, essential oils and fragrance compounds have long been widely used for the treatments of various psychological and physical disorders such as headaches, pain, insomnia, eczema, stress‐induced anxiety, depression, and digestive problems (Kako et al. 2008; Kiecolt‐Glaser et al. 2008). In modern research, the pharmacological facilitation, mood elevation, expectancy, and contextual influences of fragrances continue to be revealed through ongoing studies.

This study further confirms the effectiveness of fragrance in the cognitive domain within a driving environment. At the same time, it is important not to overlook the fact that an odorant may affect not only cognition but also emotions and even overall bodily functions. Emotions play a crucial role in driving. The study reported that sad driving is as bad as angry driving, both of them can pose higher road risks (Jeon 2016). Additionally, there is a strong connection between olfaction and motor control also regarding respiratory‐independent muscles, influencing adaptive motor outputs (Schaefer et al. 2021) and exercise performance (Meamarbashi and Rajabi 2022). There is a substantial body of evidence indicating that pleasant odors can enhance the results of physical activity, including handgrip (Cournoyer et al. 2024). Given that pleasant odors stimulate olfactory activities (Ferdenzi et al. 2015), the aforementioned effects could establish a beneficial cycle of positive reinforcement.

Equally importantly, the negative aspects of fragrance in vehicle primarily revolve around allergens and dermatitis (Perper et al. 2017; Steinemann et al. 2020), and there is less evidence to suggest that fragrances can lead to driver distraction. All of these demonstrate the potential of fragrance application in daily driving.

Nowadays, the industrial application of fragrances has reached unprecedented levels of development, making it increasingly feasible to create more favored and pleasant scents. Clearly, blended fragrances offer greater potential for personalization. Especially within the confined and limited space of a vehicle, fragrances can have the maximum impact. Importantly, this approach feels natural and pleasant, especially popular among women. This is why we believe it holds a distinct advantage for daily driving, as who would refuse the opportunity to experience “Driving with Fragrances?”.

### 4.5 Limitations and Future Work

In terms of the limitations of this study, it is important to note that the participants’ subjective evaluation of the experimental fragrance was generally positive, with a consensus that the fragrance was pleasant, relaxing, and enjoyable. As previously mentioned, a substantial body of research indicates that pleasant odors have a significant cognitive enhancing effect. While no one would intentionally introduce unpleasant odors in a car, the perception of pleasantness is inherently subjective. However, it is worth noting that this study did not include neutral or negative odors. Consequently, this study does not explore whether such significant positive effects would persist in the case of mixed fragrances receiving low subjective ratings.

Additionally, driving is a coordinated task involving the whole body, primarily engaging the visual and motor systems. This study focused solely on specific visual cognitive aspects. The results of this study demonstrate that the fatigue induced solely by driving visual simulation is sufficient to cause significantly negative brain and cognitive responses. Although it is anticipated that integrating cognitive tasks would further increase cognitive load, this remains a topic worthy of further exploration.

Furthermore, due to the complexity of the mixed fragrance itself, including variations in formulation, composition, and production process, as well as the effect of olfactory stimulation of isomeric compounds (Sowndhararajan et al. 2015), differences in experimental results may occur. A more detailed investigation based on precise formulation and chemical odor detection methods would be necessary.

## 5 Conclusion

Driving has become one of the most pervasive and routine modern activities. It is also one of the most representative factors posing risks to personal safety and property. Among all contributing factors to the accident, fatigue and sleepiness on wheel are the most common reasons, faced with inevitable uncertainties in road conditions and one's own driving state. This empirical study demonstrates the potential for improving this situation through vehicle blended fragrances. Additionally, it highlights the role of often overlooked olfactory functions in cognitive tasks and expands the application of aromatherapy in daily life.

## Author Contributions

**Tan Li**: conceptualization, resources, writing–original draft, validation, project administration. **Hua Sun**: investigation, data curation, formal analysis, resources. **Mianjie Wang**: writing–original draft, visualization, formal analysis, software, investigation. **Weihui Dai**: methodology, supervision, funding acquisition. **Xuesheng Qian**: conceptualization, methodology, writing–review & editing, project administration, supervision, validation.

### Peer Review

The peer review history for this article is available at https://publons.com/publon/10.1002/brb3.70082.

## Data Availability

The data that support the findings of this study are available from the corresponding author upon reasonable request.
